# Inferring Strain Mixture within Clinical *Plasmodium falciparum* Isolates from Genomic Sequence Data

**DOI:** 10.1371/journal.pcbi.1004824

**Published:** 2016-06-30

**Authors:** John D. O’Brien, Zamin Iqbal, Jason Wendler, Lucas Amenga-Etego

**Affiliations:** 1 Mathematics Department, Bowdoin College, Brunswick, Maine, United States of America; 2 Wellcome Trust Centre for Human Genetics, University of Oxford, Oxford, Oxfordshire, United Kingdom; 3 Pacific Northwest National Laboratory, Richland, Washington, United States of America; 4 Navrongo Health Research Centre, Navrongo, Upper East Region, Ghana; Temple University, UNITED STATES

## Abstract

We present a rigorous statistical model that infers the structure of *P. falciparum* mixtures—including the number of strains present, their proportion within the samples, and the amount of unexplained mixture—using whole genome sequence (WGS) data. Applied to simulation data, artificial laboratory mixtures, and field samples, the model provides reasonable inference with as few as 10 reads or 50 SNPs and works efficiently even with much larger data sets. Source code and example data for the model are provided in an open source fashion. We discuss the possible uses of this model as a window into within-host selection for clinical and epidemiological studies.

## Author Summary

Since the 1960’s researchers have observed that *Plasmodium falciparum* infections, the cause of most severe malaria, are frequently composed of several different strains of the parasite. In this work, the authors use Bayesian methods on whole genome sequence data to model the structure of these mixtures. Results from this method are consistent with previous approaches but provide finer resolution of these mixtures. As whole genome data in malaria research becomes increasingly common, this work will allow researchers to delve further into the within-host dynamics of the parasite, a much-discussed but previously difficult-to-access aspect of this disease.

This is a *PLOS Computational Biology* Methods paper.

## Introduction

The protozoan parasite *Plasmodium falciparum* (Pf) is the cause of the vast majority of fatal malaria cases, killing at least half a million people a year [1–3]. The parasite’s ability to develop resistance to drugs and the rapid spread of that resistance across geographically-separated populations presents a constant threat to international control efforts [4–6]. While research has elucidated many genetic factors this process, much of the genetic epidemiology of the parasite—including the effective recombination rate and the rate of gene flow across populations—is still unclear [5, 7, 8].

Understanding the implications of multiplicity of infection (MOI), where multiple strains appear to be present within a single patient’s bloodstream, is a long-standing challenge [9–13]. While MOI-focused studies implicate MOI levels with a range of conditions, including clinical severity [14], age-specific severity [15–18], parasitemia levels during pregnancy [19], and other effects [20–23], there is no broad consensus about its role in controlling the course of an infection. Still, a wide variety of studies and genetic assays—most commonly through typing the *MSP* genes—show MOI as a regular feature of clinical Pf isolates [24–26].

WGS technologies applied to Pf extracted directly from infected patients’ bloodstreams provide an unprecedented window into the structure of genetic mixture within samples [27, 28]. Initial work on this data shifted focus from estimating MOI to analysis based on inbreeding coefficients [13, 29–31]. These metrics, a form of *F*-statistic, give an estimate of the departure of within-sample allele frequencies from those expected under a Hardy-Weinberg-type equilibrium with the ambient population. From this perspective, each patient’s bloodstream is seen as a subpopulation comprised of an admixture of all strains in the local environment, ranging from a perfectly random sampling of all nearby strains (panmixia) to the repeated sampling of just a single strain (unmixed).

The initial study applying WGS to clinical Pf isolates from eight countries on three continents showed the parasite to exhibit significant population structure at continental scales, with the amount of subpopulation structure varying significantly among regions [27]. Employing an F-statistic approach to measure the inbreeding coefficient from thousands of single nucleotide polymorphisms (SNPs), this work also argued that the degree of mixture varies significantly across populations, with highly mixed samples occurring relatively frequently in west Africa but only occasionally in Papua New Guinea. The authors suggest an association between increased levels of observed mixture and increased transmission intensity in the local environment. Transmission intensity, the rate at which individuals are infected with Pf, likely determines some part of the frequency of out-crossing within parasite populations and so may be critical to understanding gene flow and strategies for resistance control [32].

In this paper, we present a statistically rigorous model that synthesizes these two distinct and previously disparate approaches to analyzing Pf clinical mixtures: assessing the number of distinct genetic types within a sample (the MOI approach [31]) and measuring the degree of panmixia with respect to the local population (the panmixia approach [33]). The model makes two significant innovations: first, a reversible jump Markov Chain Monte Carlo (MCMC) implementation to capture uncertainty in the number of strains, and second the inclusion of a panmixia term to deal with unexplained variation in the mixture. This work possesses similarities in character to the COIL algorithm [34], but can capture more complex mixture structure and is geared toward analyzing WGS data (>1000 SNPs) rather than a small number of SNPs (∼50 SNPs).

**Fig 1:**
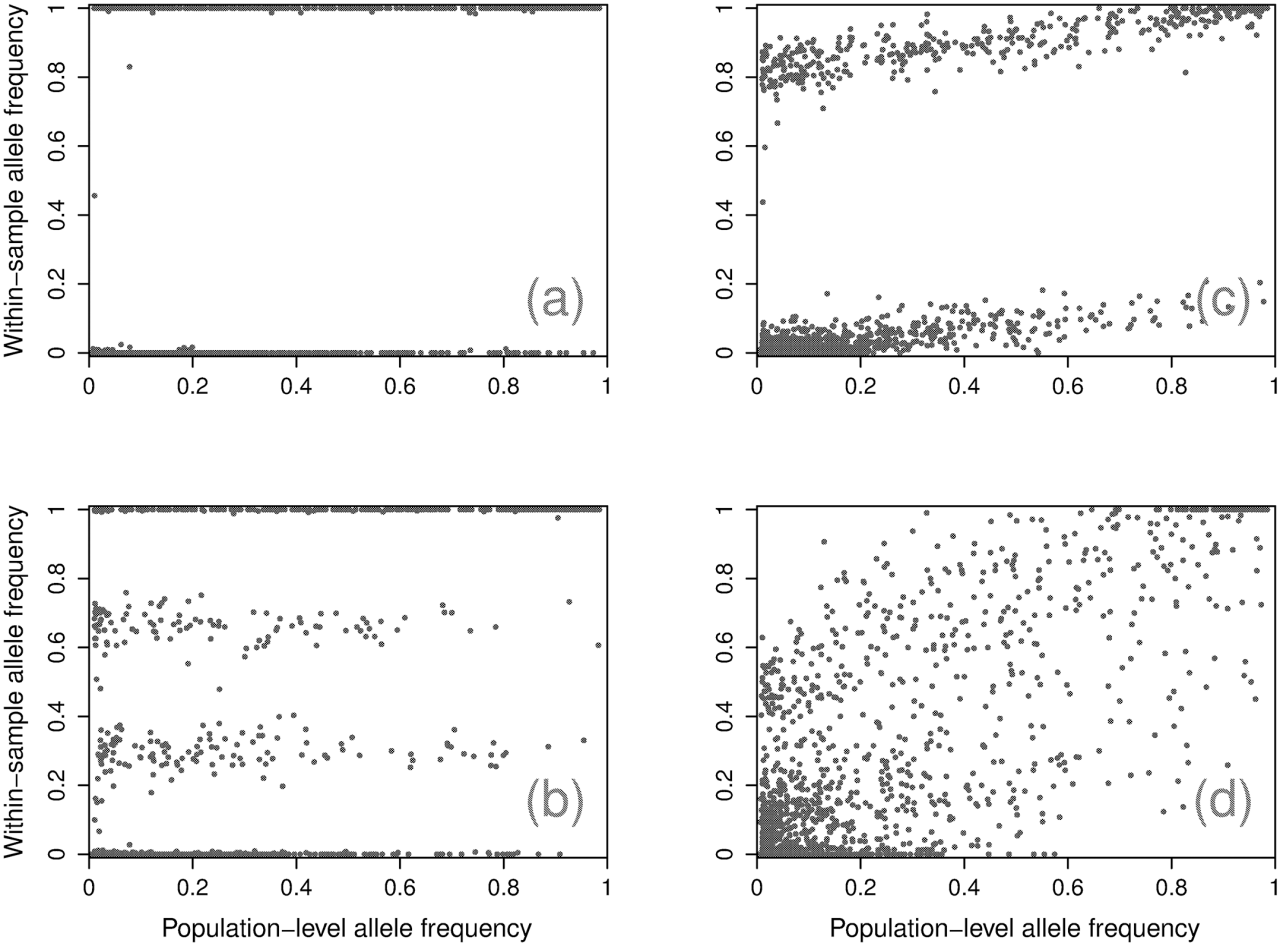
Example samples. Four representative samples with WSAF for each SNP plotted against the PLAF, showing an absence of mixture (a), a partially panmixed sample (b), a simple mixture (c), and a complex mixture (d).

This model centers around how the two sub-models—MOI and panmixia—contribute to the observed *within-sample* non-reference allele frequencies (WSAF) as they relate to the *population-level* non-reference allele frequencies (PLAF). For clarity, we will deprecate the use of *non-reference* in front of the term allele frequency, since they are all calibrated in this fashion. We will use the acronyms WSAF to denote the within-sample allele frequency and PLAF to denote population-level allele frequency to avoid confusion about the particularly allele frequency being indicated. The goal of the model is to explain observed ‘bands’ that emerge when examining SNPs WSAF as a function of their PLAF (Fig 1).

The model assumes (1) that the number of bands is a consequence of the number of distinct strains present within a sample, (2) that SNPs are unlinked, and (3) that unexplained variation is assumed to be due to a small fraction of genomes sampled under panmixia. To distinguish from an inbreeding coefficient—a similar but distinct concept—we refer to this fraction as a panmixia coefficient. The collection of WSAF bands then appears as a function of the finite mixture of the strains, with the slope in WSAF bands with respect to the PLAF explained by both the SNP distribution and the panmixia coefficient.

**Fig 2:**
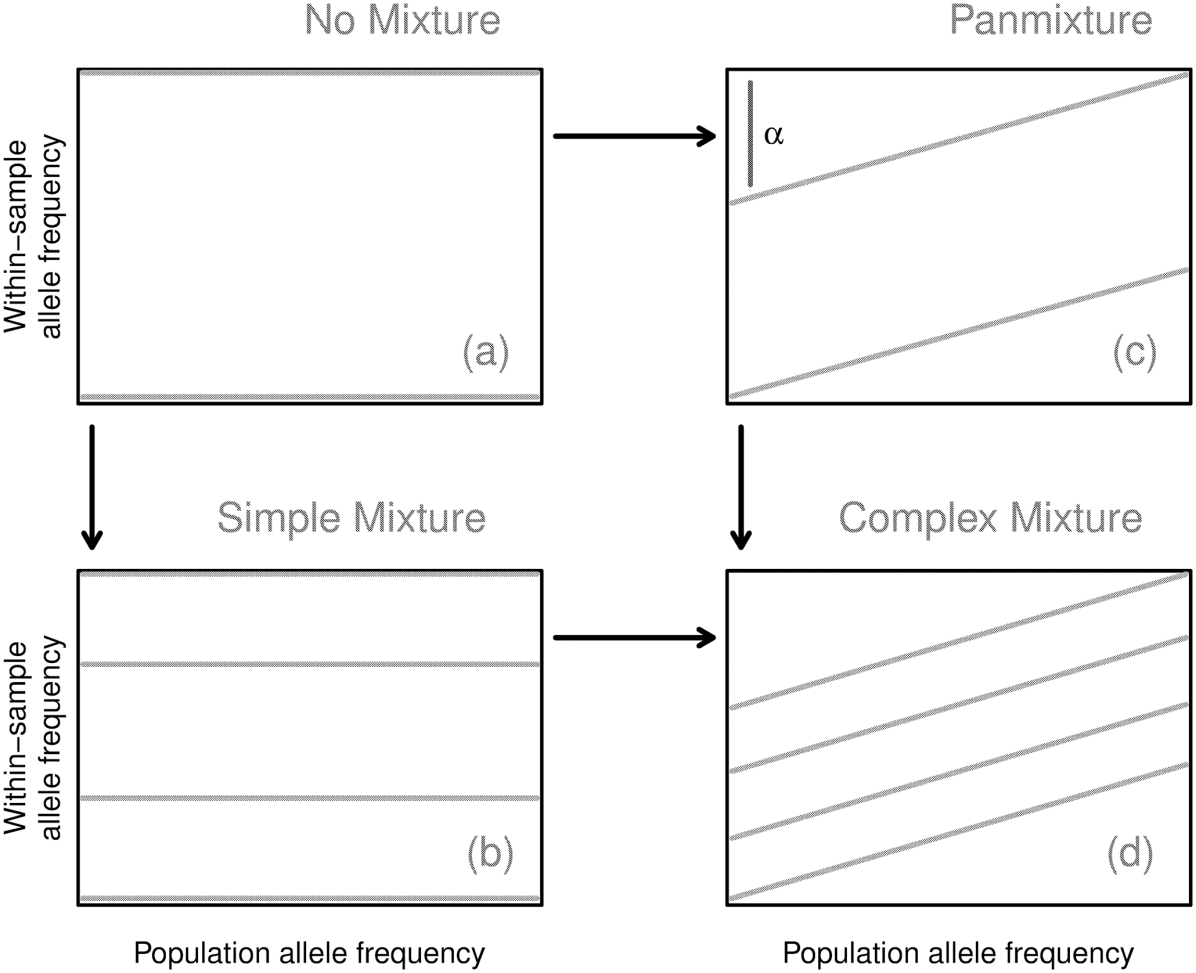
Model diagram. The structure of the model can be understood in terms of four related states connecting the WSAF to the PLAF: no mixture (upper left); simple mixture (lower left); panmixture (upper right); and complex mixture (lower right).*α* is exaggerated for explanation; realistic values are less than 0.05.

Fig 2 lays out how the consequent banding patterns can arise. In the simplest case, a sample is composed of a single, unmixed strain, and all SNPs exhibit a WSAF of zero or one (see Fig 2(a)), based on whether they agree with the reference. Consequently, the WSAF is independent of PLAF, leading to two flat bands at these values. We call these samples unmixed, since this is how a single strain with some divergence from the reference will appear. In the case where there are a finite number of strains mixed within a sample, then at a given SNP position some number of the strains will exhibit a reference allele and some a non-reference allele. The WSAF for that SNP is determined by the proportions of non-reference strains in the sample mixture. Observing many SNPs displays ‘bands’ of constant WSAF across the PLAF. Thus, for *K* component strains there are 2^*K*^ possible combinations of biallelic states, leading to that number of bands.

A fraction of the Pf organisms present within the blood may not be from any of the dominant strains. We model these as randomly sampled from the local population according to simple panmixia. Observationally, this leads to a consistent change in the slope of each of the bands. To see this, consider an admixture of two distinct Pf populations: a single strain, representing 1 − *α* of the within-sample genomes, and the remaining *α* that we assume follow panmixia. The *α* tilt in the WSAF arises from the fact that for this proportion of organisms the probability of sampling non-reference allele is proportional to the PLAF (Figs 1(c) and 2(c)). Samples with high *K* appear to have additional tilt due to the higher probability of non-reference alleles occurring at high PLAF (Figs 1(d) and 2(d)).

The paper proceeds as follows. We first detail the structure of the WGS data, introduce some notation, and the essential mathematical structure of the model. We then present an extensive simulation study on the performance of the model, an application of the model to artificial laboratory mixtures, and an examination of its application to field isolates collected from northern Ghana. We conclude by discussing the strengths and weaknesses of the model, a means of experimental validation, and potential consequences for the etiology of clinical malaria.

## Materials and Methods

### Data

The field WGS data come from Illumina HiSeq sequencing applied to Pf extracted from 419 clinical blood samples collected from infected patients in the Kassena-Nankana district (KND) region of Upper East Region of northern Ghana. Collection occurred over approximately 2 years, from June 2009-June 2011. The raw sequence reads for these data are accessible through the PF3K project https://www.malariagen.net/projects/parasite/pf3k. This includes data from the MalariaGEN Plasmodium falciparum Community Project on www.malariagen.net/projects/parasite/pf. On the website for this method, we provide read count data subsampled from the full data set. The artificial laboratory samples were sequenced and called per protocols given in [35]. The raw sequence data is available through the European Nucleotide Archive with the accessions available in the S1 Text.

The full sequencing protocol and collection regime are described in [27]. After quality control measures, all samples were examined, and following a documented protocol comparing against world-wide variation, 198,181 single-nucleotide polymorphisms (SNPs) were called [27]. These are exclusively coding SNPs found outside of the telomeric and subtelomeric regions that exhibit unusual structural properties. Each SNP xcall provides the number of reference and non-reference read counts observed at each variant position within the genome, ascertained against the the 3D7 reference [36]. These data were exhaustively examined for spurious heterozygosity and evidence of DNA contamination, with mixed calls verified using time-of-flight mass spectrometry at greater than 99% accuracy [27].

For this project, we further filtered the data. First, multiallelic positions were reclassed as biallelic. We then excluded positions that exhibited no variation within the KND samples, any level of missingness (no read counts observed), or minor allele frequency less than 0.01. To remove low quality samples, we removed those with more than 4,000 SNPs missing and fewer than 20 read counts, following an inflection point observed in S1 Fig. These cleaning measures left 2,429 SNPs in 168 samples. These SNPs exhibit desirable properties for model inference—high and consistent coverage across all samples—that could likely be expanded to non-coding or less stringent cleaning standards without issue. More than 95% of the remaining samples’ sequencing was completed without PCR amplification. We observe little apparent population structure among the samples, evidenced either by principal components analysis or a neighbor-joining tree of the pairwise difference among samples (S2 Fig). The data preparation scripts are available with the source code for the model, https://github.com/jacobian1980/pfmix/.

### Notation

Following the data preparation and cleaning, our analysis begins with a set of *N* = 168 clinical samples, each composed of *M* = 2,429 SNPs. At each SNP *j* within each clinical sample *i*, we observe *r*_*ij*_ reads that agree with the reference genome and *n*_*ij*_ reads that do not agree. The total number of read counts in sample *i* at SNP *j* is then *n*_*ij*_ + *r*_*ij*_. For a sample *i*, we write the complete data across all SNPs as $D_{i}=\left[\left(r_{i1},n_{i1}\right),\cdots ,\left(r_{iM},n_{iM}\right)\right]$. For each SNP *j*, we associate a PLAF *p*_*j*_. The collection of all *p*_*j*_ we refer to as P.

Conditional upon the number of strains *K*, there are 2^*K*^ bands, indexed by *r* = 1, ⋯, 2^*K*^. The full collection of bands we call Q, with *q*_*ijr*_ indicating the WSAF for sample *i* at SNP *j* in band *r*. The probability of a SNP lying within the distinct bands across the PLAF is specified by a mixture component *λ*_*r*_, which is a function of the PLAF detailed below. The degree of panmixia in a sample is given by *α*, a value between zero and one. A complete list of the model parameters is given in Table 1.

**Table 1:** Parameters and definitions for the model and its description.

Parameter	Definition
*N*	Number of samples
*M*	Number of SNPs
*K*	Number of strains
*i* = 1, ⋯, *N*	Index for samples
*j* = 1, ⋯, *M*	Index for SNPs
*r* = 1, ⋯2^*K*^	Index for bands / strain mixtures
*p*_*j*_	(Non-reference) allele frequency for SNP *j*
$P=[p_{j}]$	The PLAF for all SNPs
$Q=[q_{ij}]$	Within-sample allele frequency for SNP *j* in sample *i*
*α*	Degree of panmixia within a sample, panmixia coefficient
$S=[s_{1},\cdots ,s_{K}]$	Strains in a sample
$W=[w_{1},\cdots ,w_{K}]$	Strain proportions in a sample
*λ*_*r*_	Band proportions within sample
*ν*	Variation parameter for Beta-binomial
WSAF	Within-sample allele frequency
PLAF	Population-level allele frequency

### Model

Statistically, the model takes the form of a finite mixture model with the mixture components associated with individual bands [37, 38]. We take a Bayesian approach to inference and construct the model by giving an overall rationale for the decomposition of the posterior distribution, and then justify the appropriate choice of probability distributions for each of the terms [39].

#### Decomposition

We assume that samples are independent of each other and that the SNP data for each sample depends solely on the number of bands (*K*), the WSAF (Q), the PLAF (P), and a shape parameter *ν*. As samples are treated independently, we deprecate sample-specific subscripts for the model parameters. Considering the data for a single sample, $D_{i}$, the posterior distribution can then be written as:

$$\begin{array}{ccc} P(Q,P,W,\alpha ,\nu ,K|D_{i}) & \propto & P\left(D_{i}|Q,P,W,\alpha ,\nu ,K\right)\cdot P\left(Q,P,W,\alpha ,\nu ,K\right)\\ & = & P\left(D_{i}|Q,P,\nu ,K\right)\cdot P\left(Q,P,\nu ,K,W,\alpha \right). \end{array}$$ (1)

We also assume that the WSAF depends only on the PLAF, the panmixia coefficient, the number of strains, and their proportions within the sample, allowing the right-hand side of Eq (1) to be further decomposed, by noting that:

$$\begin{array}{ccc} P(Q,P,\nu ,K,W,\alpha ) & = & P(Q|P,\nu ,K,W,\alpha )\cdot P(P,\nu ,K,W,\alpha ). \end{array}$$ (2)

While the strain proportions clearly depend on the number of strains, the remaining parameters we take to be independent of this value and of each other. This means that the last right-hand side term in Eq (2) becomes:

$$\begin{array}{ccc} P(P,\nu ,K,W,\alpha ) & = & P(P)\cdot P(\nu )\cdot P(W|K)\cdot P(K)\cdot P(\alpha ). \end{array}$$ (3)

Substituting Eqs (2) and (3) into Eq (1), yields the final decomposition:

$$\begin{array}{ccc} P(Q,P,W,\alpha ,\nu ,K|D_{i}) & \propto & P\left(D_{i}|Q,P,\nu ,K\right)\cdot P\left(Q|P,\nu ,K,W,\alpha \right)\cdot \\ & & \;P(P)\cdot P(\nu )\cdot P(W|K)\cdot P(K)\cdot P(\alpha ). \end{array}$$ (4)

We now specify each of the terms on the right-hand side above as probability distributions.

#### Likelihood: $P(D_{i}|Q,P,\nu ,K)$

Within band *r*, the WSAF at SNP *j* in sample *i* is *q*_*ijr*_. Supposing that read counts at *j* are identically and independently distributed with probability *q*_*ijr*_, we model the probability of the data (*r*_*ij*_, *n*_*ij*_) as a Beta-binomial distribution, allowing us to fit greater dispersion than expected under a pure binomial. We parameterize this distribution in terms of *q*_*ijr*_ and *ν* rather than the more commonly used shape and scale parameters, *α* and *β*, with the relationship *q*_*ijr*_ ⋅ *ν* = *α* and (1 − *q*_*ijr*_) ⋅ *ν* = *β*. This parameterization allows us to write the model in terms of the allele frequency that defines each band. The additional *ν* is a shape parameter that serves as an over-dispersion parameter. These give a likelihood expression for a SNP within a band as:

$$\begin{array}{ccc} P(n_{ij},r_{ij}|r,q_{ijr},\nu ) & = & \left(\begin{array}{c} n_{ij}+r_{ij}\\ n_{ij} \end{array}\right)\cdot \frac{\text{B}(n_{ij}+q_{ijr}\cdot \nu ,r_{ij}+\left(1-q_{ijr}\right)\cdot \nu )}{\text{B}(q_{ijr}\cdot \nu ,\left(1-q_{ijr}\right)\cdot \nu )}, \end{array}$$ (5)

where B is the beta function.

As any SNP could lie within any band, we employ a novel version of the finite mixture model to capture this segregation. Given *K* strains, there are then 2^*K*^ ways that the strains can be assorted into non-reference and reference allele states at any given position *j*. A given band *r* arises from *C*_*r*_ strains exhibiting the non-reference allele and 2^*K*^ − *C*_*r*_ strains exhibiting the reference allele. Supposing no population structure among the strains and neglecting linkage among SNPs, the probability that a given SNP will be in that band is simply the probability of drawing *C*_*r*_ non-reference alleles and 2^*K*^ − *C*_*r*_ reference alleles, conditional upon *p*_*j*_:

$$\begin{array}{ccc} P(\text{SNP}\;j\in \text{band}\;r|p_{j}) & = & p_{j}^{C_{r}}\cdot {\left(1-p_{j}\right)}^{2^{K}-C_{r}}\\ & = & \lambda_{r}\left(p_{j}\right). \end{array}$$

Consequently, the density of the mixture coefficients for each band varies across the PLAF but such that they always sum to 1 across all bands at any SNP position *j*. This gives a likelihood across all bands as:

$$\begin{array}{ccc} P(D_{ij}|Q,P,\nu ,K) & = & \sum_{r=1}^{2^{K}}P\left(\text{SNP}\;j\in \text{band}\;r|p_{j}\right)\cdot P\left(n_{ij},r_{ij}|r,q_{ijr},\nu \right)\\ & = & \sum_{r=1}^{2^{K}}\lambda_{r}\left(p_{j}\right)\cdot P\left(n_{ij},r_{ij}|r,q_{ijr},\nu \right). \end{array}$$

Following from the assumption of no linkage, SNPs will independently assort into bands. This leads to a product-sum form for the likelihood for $D_{i}$:

$$\begin{array}{ccc} P(D_{i}|Q,P,\nu ,K) & = & \prod_{j=1}^{M}[\sum_{r=1}^{2^{K}}\lambda_{r}\left(p_{j}\right)\cdot P\left(n_{ij},r_{ij}|r,q_{ijr},\nu \right)]. \end{array}$$ (6)

#### Band structure: P(Q|P,ν,K,W,α)

The complex mixture model contains two distinct subcomponents that we call the simple mixture model and the panmixture model, respectively. Both models generalize the unmixed case, though in different ways. We first characterize the unmixed model and the two extensions before showing how these can be combined to create the complex model. In practice, we only fit data using the full model and allow it to indicate the number of strains, their proportions, and the degree of panmixia. We do not know the number of strains *a priori* so we employ a reversible jump approach to infer the posterior distribution on *K*. However, for the purpose of detailing the model, we assume that *K* is known.

*Unmixed model*. In an unmixed sample only one strain is present and the panmixture coefficient is zero (i.e. *K* = 1 and *α* = 0). Consequently, all SNPs exhibit a WSAF of either zero or one (Fig 2(a)). There are then two bands, *r* = 1, 2 and *q*_*ij*1_ = 0 and *q*_*ij*2_ = 1.

*Simple mixture model*. Conditional upon *K*, the distinct strains, *s*_1_, ⋯, *s*_*K*_, are combined together in the sample with proportions, $W=(w_{1},\cdots ,w_{K})$, but that *α* = 0. Necessarily, ∑_*k*_ *w*_*k*_ = 1. For each SNP *j*, the probability of being within band *r* is given by *λ*_*r*_(*p*_*j*_), as above. Band *r* is defined by a vector *v*_*r*_ = (**1**_{*s*_1_ ∈ *r*}_, ⋯, **1**_{*s*_*K*_ ∈ *r*}_), where **1**_{*s*_*k*_ ∈ *r*}_ is a function indicator function on whether strain *k* exhibits a non-reference allele within the sample. The WSAF of band *r* for SNP *j* (*q*_*ijr*_) is then given by the sum of proportions for strains that exhibit a non-reference allele:

$$\begin{array}{ccc} q_{ijr} & = & \sum_{k=1}^{K}w_{k}\cdot 1_{\left\{s_{k}\in r\right\}}. \end{array}$$ (7)

Taken across all *r* bands, this leads to 2^*K*^ bands with zero slope and corresponding proportions (0, *w*_1_, ⋯, *w*_*K*_, *w*_1_ + *w*_2_, *w*_1_ + *w*_3_, ⋯, 1).

*Panmixture model*. In the simplest case, the panmixture model represents the admixture of two distinct Pf populations when *K* = 1: a single strain, representing 1 − *α* of the within-sample genomes, and a random sample of alleles from the local population for the remaining *α* genomes. *α* can be considered the fraction of unexplained variation in the sample. When *α* = 0 the model reduces to the unmixed case (see Figs 1(b) and 2(b)). For each position *j*, there are still only two bands: the higher one corresponding to the non-reference allele being present in the dominant strain, and the lower one corresponding to its absence. However, the WSAF for these bands varies according to *p*_*j*_ with slope *α*. To resolve *q*_*ijr*_, first consider the upper band, *r* = 2. At any position *j*, 1 − *α* of the reads come from the dominant strain. The remaining reads, each sampled randomly from the local population, each have probability *p*_*j*_ of being non-reference. This leads to *q*_*ij*2_ = (1 − *α*) + *α* ⋅ *p*_*j*_. For the lower band, the dominant strain contributes no non-reference reads so *q*_*ij*1_ = *α* ⋅ *p*_*j*_.

*Complex mixture model*. The complex model synthesizes the simple mixture and panmixture models so that both *K* and *α* may vary. In this case, at position *j*, *α* of the reads are sampled randomly from the across the local population, contributing a fraction of *α* ⋅ *p*_*j*_ non-reference alleles. The state of the remaining reads are determined by W as in Eq (7). For band *r* at position *j*, the WSAF is then given by:

$$\begin{array}{ccc} q_{ijr} & = & \left(1-\alpha \right)\cdot (\sum_{k=1}^{K}w_{k}\cdot 1_{\left\{s_{k}\in r\right\}})+\alpha \cdot p_{j}. \end{array}$$ (8)

There are then 2^*K*^ bands with proportions (0, *w*_1_, ⋯, *w*_*K*_, *w*_1_ + *w*_2_, *w*_1_ + *w*_3_, ⋯, 1) and slope *α*.

#### Priors

For the remaining four probability distributions we place the following vague prior distributions:

$$\begin{array}{ccc} W|K & ∼ & \text{DIRICHLET}(1_{K})\\ \alpha & ∼ & \text{UNIFORM}(0,1)\\ \nu & ∼ & \text{EXPONENTIAL}(5)\\ K & ∼ & \text{zero-truncated}\;\text{POISSON}(2), \end{array}$$

where **1**_*K*_ is a vector of *K* ones.

### Inference

We infer the model parameters using a standard reversible jump MCMC approach [40, 41] with one exception: we first calculate maximum-likelihood estimates (MLE) for P across all samples and then treat these as fixed when inferring the remaining parameters [42]. This choice is motivated by statistical expedience and computational speed: except for P, the parameters of the model are independent across samples and so this approximation enables the algorithm to infer parameters in parallel rather than jointly. This avoids the difficulties of performing inference on the number of strains within all samples simultaneously. Running in parallel also increases the computational speed of the implementation by at least an order of magnitude. Since the sample collection is large enough that the PLAF is nearly independent of any given sample, we do not expect this approximation to significantly bias inference.

For each SNP *j*, the MLE derives from treating the non- and reference reads within a sample as coming from a binomial distribution with parameter *p*_*j*_. This leads to:

$$\begin{array}{ccc} {\hat{p}}_{j} & = & \sum_{i}^{N}n_{ij}/\sum_{i}^{N}\left(n_{ij}+r_{ij}\right). \end{array}$$

To infer the number of strains, *K*, for each sample, we employ a pair of complementary split/merge reversible jump MCMC moves. To specify the split move first not that in moving from *K* → *K* + 1 that the transformation only affects the parameter W. If we randomly select *w*_*k*_, 1 ≤ *k* ≤ *K*, then we can split this into two components, *u* ⋅ *w*_*k*_ and (1 − *u*) ⋅ *w*_*k*_, where *u* is drawn from a uniform distribution. This establishes a diffeomorphism between parameters at *K* and *K* + 1 with Jacobian *w*_*k*_. The proposal ratio is (*K*^2^ − *K*)/*K* = *K* − 1. The acceptance ratio then is the product of the proposal ratio, Jacobian, the likelihood ratio, and the prior likelihood. The merge move randomly selects two states, *k*_1_ and *k*_2_, and merges them to *k*′ by setting *w*′ = *w*_*k*_1__ + *w*_*k*_2__. The Jacobian and proposals are the reciprocal of those for the split move.

Conditional on P and *K*, for each of the three parameters, *α*, W, and *ν*, we propose new values directly from the prior distribution. This leads to Metropolis-Hastings ratios almost solely dependent on the ratio between the likelihood and priors for the proposed state to those for the current. The inference scheme is implemented in set of scripts for the R computing language, and can be found under the Academic Free License at https://github.com/jacobian1980/pfmix/s. For a single sample with *K* = 5, a sufficiently long MCMC run takes approximately 10 minutes on a single high-performance computing core.

## Results

### Simulations under the model

To demonstrate the efficacy of the model and our implementation, we present a simulation study examining the algorithm’s performance under a range of simulated data. We consider two distinct aspects of the inference: how well the model infers the number of strains, and, conditional upon that number, how well it infers the model’s other parameters. We simulate data from the model in the following way. Conditional upon the number of SNPs (*M*), panmixture coefficient (*α*), number of strains (*K*) and the sum of the read counts (*C*) we draw a vector of probabilities (W) from a uniform Dirichlet distribution. We combine the values of W in all possible permutations to create the 2^*K*^ bands and assign the PLAF for the SNPs evenly from 1/*M* to 1, so that the $j^{\text{th}}$ SNP has PLAF $\frac{j}{M}$. For each SNP, we first probabilistically select the band it occupies according to Eq (6). We then simulate read counts from the likelihood (Eq 5) with *q*_*ijr*_ per Eq (8). For all simulations, we set *ν* = 10. We run the simulation across the range of values for *M*, *α*, *K* and *C* given in Table 2. For each parameter set, we create 10 independent realizations.

**Table 2:** Table of simulated parameter values. *C* is the number of read counts while *M*, *K* and *α* are as in Table 1.

Parameter	Values:			
M	50	150	500	2500
C	10	25	100	250
*α*	0.001	0.01	0.1	
*K*	1	3		

#### Number of components

**Fig 3:**
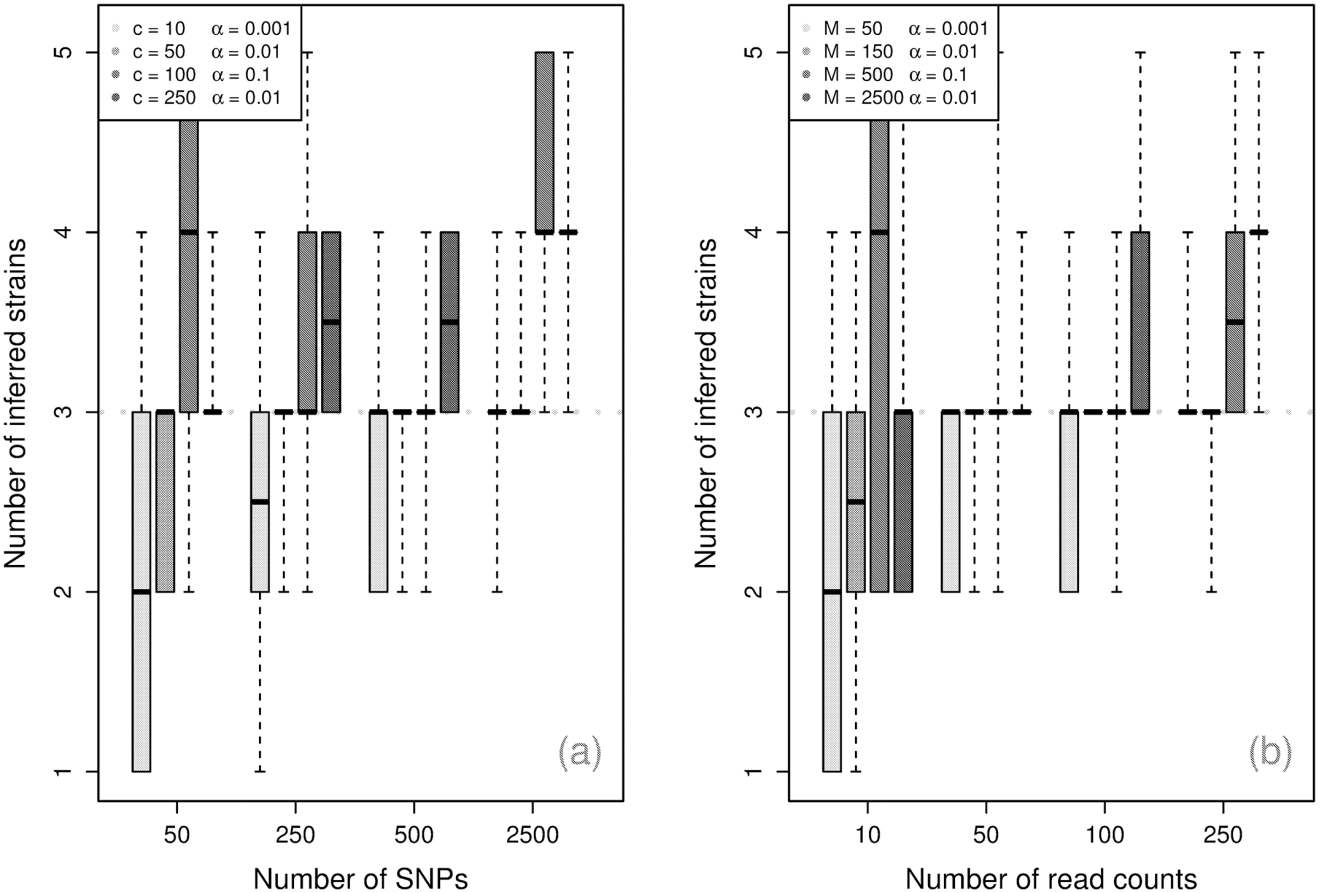
Component inference. *Maximum a posteriori* (MAP) inferred number of components by number of read counts across 10 simulations, with dotted line at the true number of components.

Fig 3 shows the algorithm’s performance for inferring the number of components becomes more accurate with the number of SNPs and the number of reads, with 50 SNPs and 25 read counts sufficient to reliably recover the simulated values. With more SNPs, the requirement on read counts can be reduced to 10 with similar performance. Conditional upon *α*, the simulations indicate that the number of SNPs is the largest determinant of performance, and the sum of the read counts playing an important supporting role. Inference of the number of strains is generally strong at low panmixture levels (small *α* values), but is noticeably more conservative for *α* = 0.1.

#### Parameters

**Fig 4:**
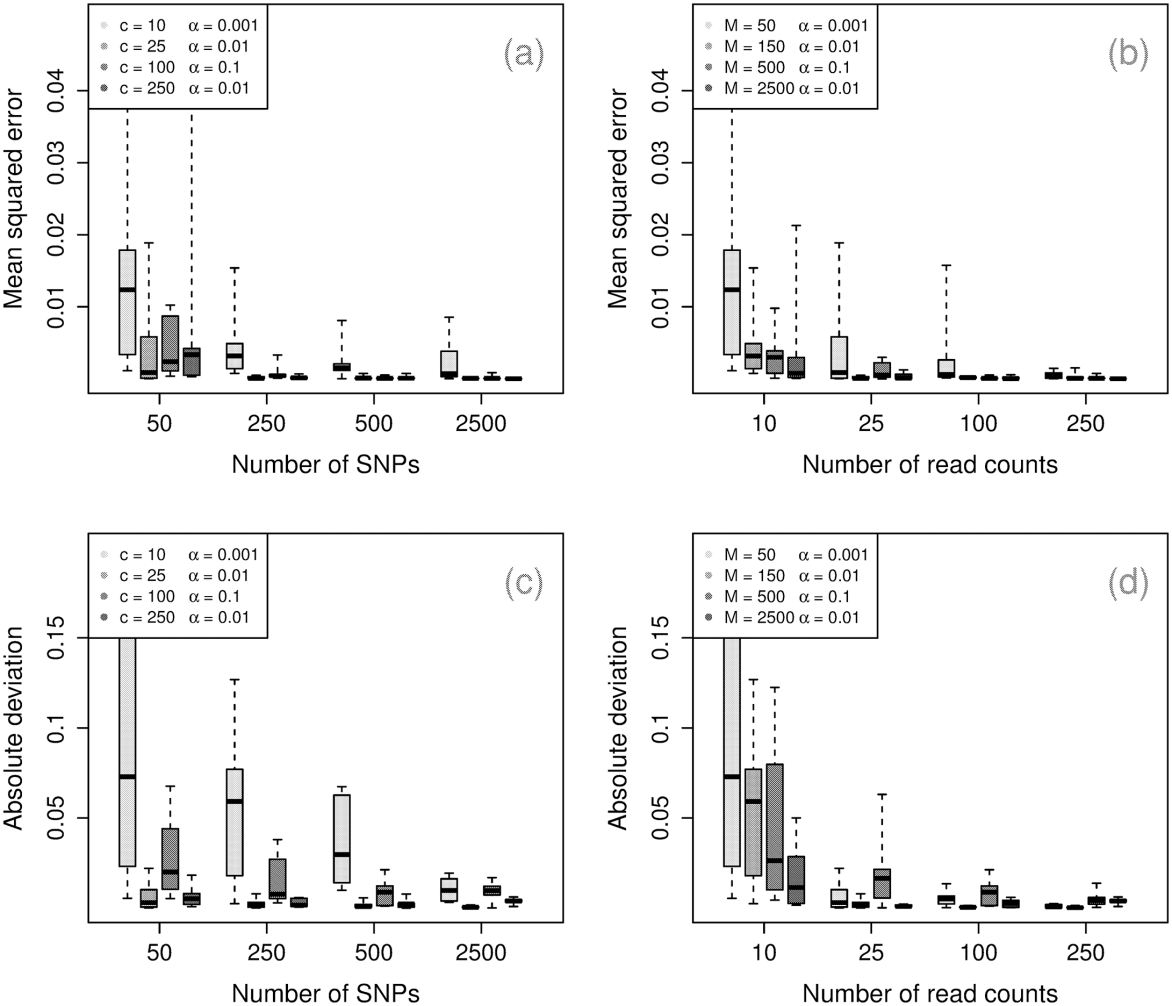
Performance for parameter inference. Upper row: mean squared deviation for strain frequencies by number of read counts (left) and by number of SNPs (right). Lower row: absolute normalized deviation for panmixia coefficient by number of read counts (left) and by number of SNPs.

Fig 4 shows similar performance for inference of the strain proportions, W, and panmixture coefficient, *α*. For W, we report the mean squared deviation. For *α*, we report the absolute normalized deviation to account for relative difference from the true value. For both parameters, we observe that the number of SNPs is the strongest determinant of accuracy, with *M* = 150 ensuring moderately strong performance. Again, high *α* moderately decreases the quality of inference for the strain proportions.

### Laboratory artificial mixtures

We apply the algorithm to 18 artificial laboratory mixtures. These artificial samples were generated by taking stock of two standard Pf lines, DD2 and 7G8, and adding them together in the fixed proportions given in S1 Table, and completing then Illumina sequencing and variant-calling with using the same protocols as [27]. Samples had a median of 65 reads for the variants considered here. Complete sequencing protocols and laboratory methods detailed in [35] (data available at European Nucleotide Archive). Both strains have high-confidence reference sequences. We subsample 2000 SNPs from the 23,109 SNPs available for comparison based on non-reference WSAF. The results in S1 Table show very strong agreement between the laboratory and inferred mixtures. The inferred *α* for all samples was less than 0.001 and had Bayes factor for non-zero *α* as less than 1, indicating that the samples have little unexplained mixture observed relative to the field samples.

### Clinical samples from northern Ghana

**Fig 5:**
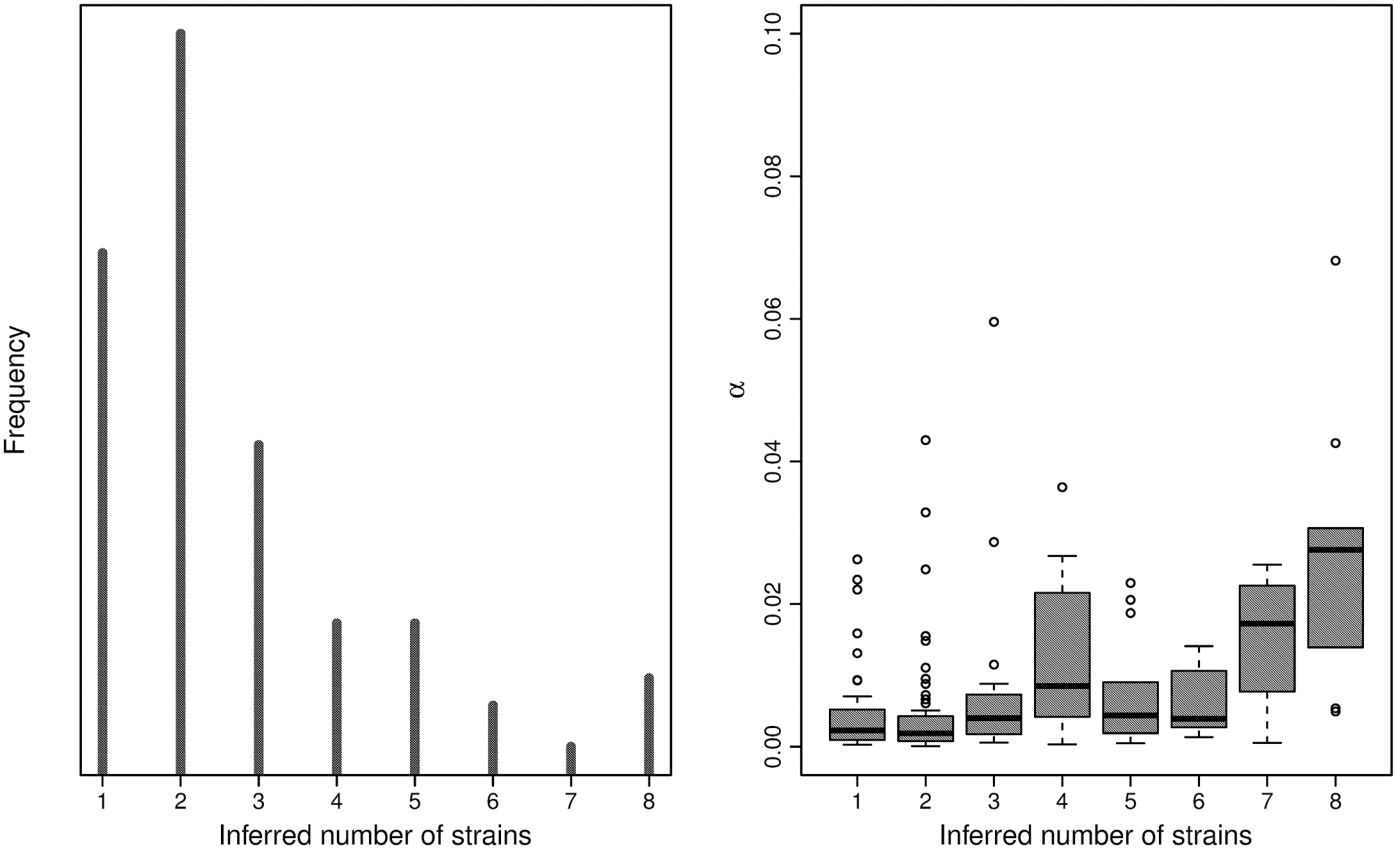
Ghanian sample summary. The frequency of inferred number of strains per sample (left) and and the panmixia coefficient by number of strains (right). MAP estimates used.

Applying the algorithm to the 168 high-quality samples from KND, we observe numbers of strains ranging from 1 to 7, with *α* falling between 0 and 0.14, and a moderate correlation between *K* and *α* (Fig 5). The largest subset of samples were unmixed, with *K* = 1 and *α* < 0.01, though the majority of samples exhibit low but noticeable levels of admixture, with *K* = 2, 3, 4 and 0.01 ≤ *α* ≤ 0.03. A small number of samples exhibit complex mixtures, with *K* > 4 and *α* typically greater than 0.02. These samples also exhibit the most variance in the posterior estimate of *K*, frequently ranging from 3 to 8. To examine the necessity of the panmixia model to capture unexplained variation in the field samples, we calculate a Bayes factor for each sample under the two models, *M*_0_: *α* = 0 and *M*_1_: *α* ≠ 0. Since this is a single parameter, we employ the Savage-Dickey ratio calculation as in [43]. We find that 78 samples give factors larger than 10, indicating strong evidence for *M*_1_, and 9 samples give factors larger than 100, indicating overwhelming evidence for *M*_1_.

**Fig 6:**
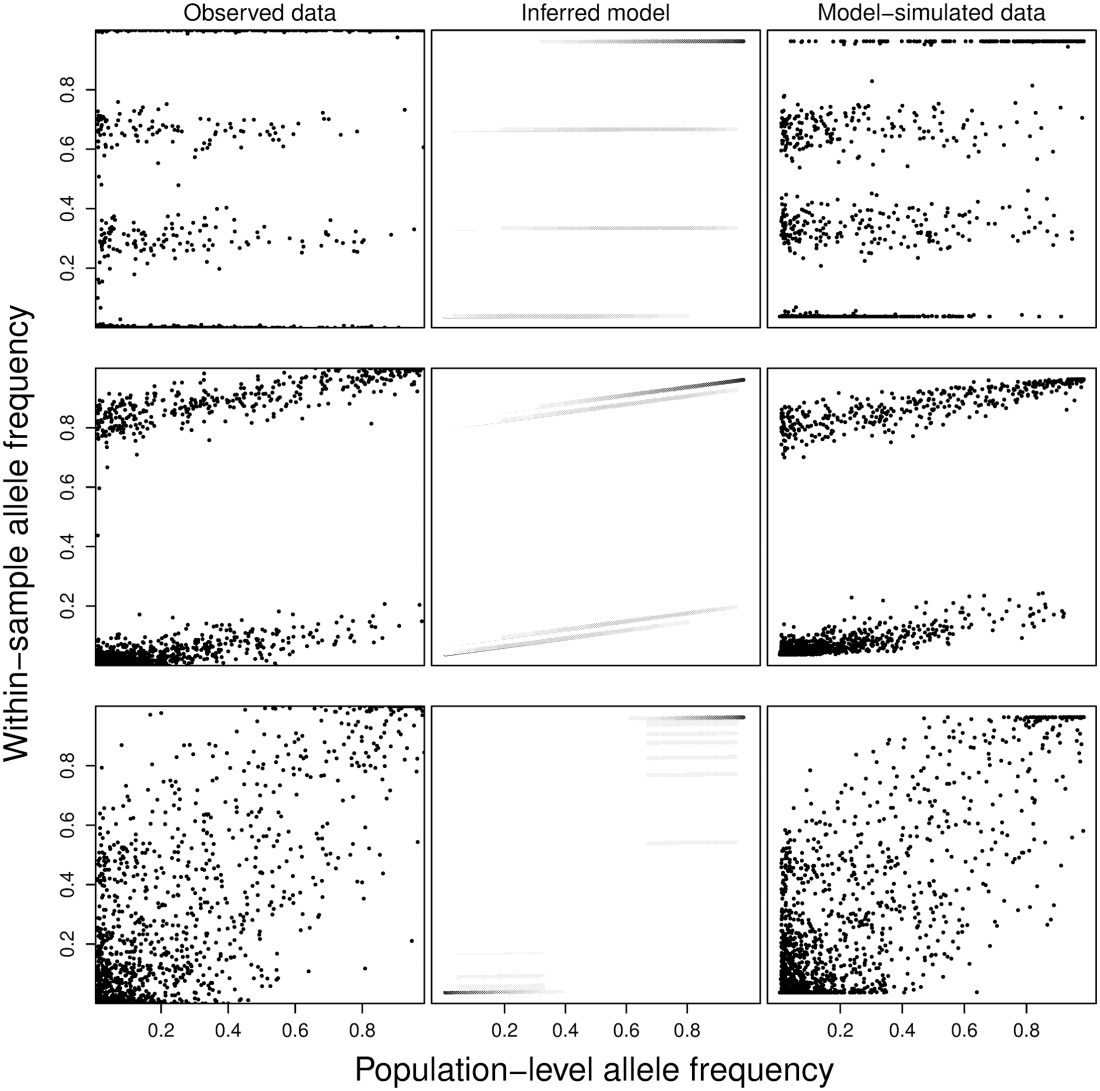
Examples of real and model-simulated data. For three samples (rows), we present the observed data WSAF plotted against the PLAF (first column), a diagram of the inferred model indicating the bands, proportions, and panmixia coefficient (second column), and data simulated under the inferred model. Panmixia coefficient and strain proportions are the MAP values. In the second column, the model’s PLAF-varying mixture densities are shown in grey scale, with black equal to one.

**Fig 7:**
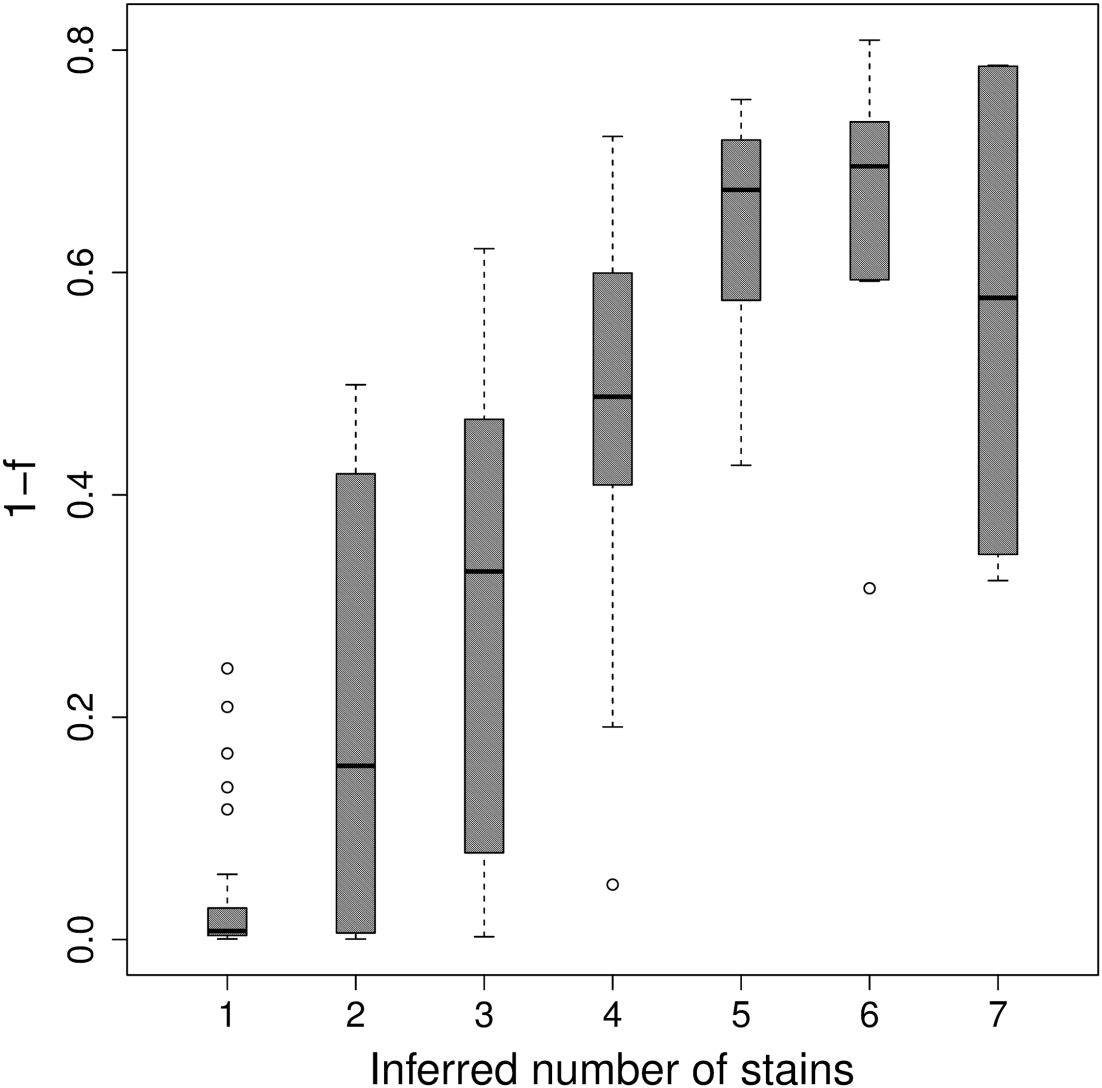
Number of strains by F-statistic. Boxplot of the inbreeding coefficient (1 − *F*_*is*_) for each sample grouped by the MAP number of inferred strains.

To visually inspect the quality of the results, we generate figures for each of the samples showing the observed WSAF and PLAF data, the inferred model structure, and data simulated under the inferred model following the observed PLAF. We show examples of these plots for three typical samples in Fig 6. Nearly all samples (158/168), across all different mixture patterns, show strong visual correspondence between the observed and model-simulated data. Samples where PCR amplification was used (9 samples) exhibit no unusual features other than low values for *α* relative to the remaining samples. We also observe a strong correlation between the inferred number of components and an estimate for the inbreeding coefficient for each sample (Fig 7) [29]. These results are consistent with the high rate of MOI previously observed in Ghanaian clinicial samples [24, 44, 45].

## Discussion

In this work we show how to infer strain mixture within Pf isolates using WGS with two improvements over previous efforts: an additional model for unexplained variation based on a panmixia and a reversible jump implementation that accounts for uncertainty in the underlying number of strains. Simulations show that the model can perform accurate inference (MSE < 0.05 for strain proportions) with as few as 50 SNPs and 10 read counts per SNP. Simulations with more than 100 SNPs or at least 25 read counts give highly accurate results (MSE < 0.02). In artificial laboratory mixtures the model provides excellent agreement with baseline mixture. In field samples the model provides strong agreement with observed data and evidence based on Bayes factors indicates that some unexplained variation is present in a significant fraction of samples.

While the method works efficiently in practice, a number of possible improvements could strengthen its statistical performance. Most immediately, creating a full Bayesian approach rather than the parallelizing implementation here—while likely not improving the parametric inference for individual samples—would provide the full posterior distribution across all samples. The panmixia model is one of several possible approaches to dealing with additional within-sample variation that rigorous model comparison could reveal. The model also does not perform haplotype phasing to resolve the sequence of the underlying strains [46–48]. The analysis here suggests that a method for estimating haplotypes would be straight-forward for some samples but difficult for others (say, when *α* is greater than 0.05). Researchers may be particularly interested in whether, in these phased samples, particular stretches of the genome appear more or less frequently in the dominant strains than others, indicating structures of immunological or environmental selection. This is a natural avenue for statistical methods development.

The model makes a number of simplifying assumptions that may be violated in practice. The model presumes that SNPs are unlinked and consequently independent for the purpose of calculating the likelihood. Given the high recombination rate of Pf this assumption may hold for the majority of pairs of SNPs, but neglects correlations that appear locally (∼ 10 kB). However, we expect that this independence assumption serves to moderately weaken the inferential power of the model rather than cause any type of bias since it effectively fails to include possibly informative data. More problematic is the model’s implicit assumption of limited population structure. In the case of the KND samples, and perhaps in much of west Africa, this assumption appears supported [27, 49]. In other contexts, specifically southeast Asia, recent population bottlenecks and selection suggest that this assumption will be violated [50]. The consequences on this model inference are unknown but may be partially resolved with appropriate simulation studies.

The model will work with any technology capable of typing multiple variants and where the measurement of the fraction of non-reference variants is unbiased. It was developed for WGS data but is not specific to the sequencing employed and should work similarly for Illumina, 454 and Pacific Bioscience read technologies. As noted in the results, we observe that the small number of field samples where PCR amplification was used did not appear unusual other than exhibiting relatively low *α* values. This is could be due to preferential amplification of the dominant strains, suggesting that PCR-based approaches may obscure some aspects of natural infections. This model is not appropriate for data from RFLP assays or DNA microarrays without substantial modification.

In principle, the model can be explicitly tested against experimental mixtures more complex than those presented above. Laboratory facilities with the capacity to store many field strains (>100) could generate artificial samples in an experimental analog of our simulation procedure. Starting with *N* unmixed strains at equal dilution, they could create mixtures by first fixing the required sequencing volume as *η*, and the desired parameters for panmixia (*α*), number of component strains (*K*), and their mixture parameters, W. For the finite mixture component, they would then combine volumes of η·W from the *K* strains. For the panmixture component, they would then fix some large number but experimentally feasible number of strains (say 50) and randomly sample from all of them a volume of *η*/50. Combining these into a final sample and applying WGS sequencing, will yield data that we hypothesize will closely follow the integrated model outlined above, with *ν* capturing the experimental variation. Naturally, consistent results would indicate the sufficiency of the model but not its necessity, holding out the possibility of a more minimal description. These results could be further compared against other next-generation technologies—such as single-cell sequencing—that have been deployed to understand Pf clinical mixtures [51].

The model presents an important new tool for interrogating the biology of clinical Pf infections. The ability to measure the structure of strain mixture connects to many aspects of Pf epidemiology including seasonality, transmission intensity, outcrossing, and rates of gene flow. It also presents a means for clarifying the poorly detailed structure of intra-host infection dynamics, such as strain selection or density-dependent selection [52], by resolving how the model parameters change within the course of an infection or in response to drug intervention. This approach can serve as a means for researchers to empirically resolve these hypotheses.

## Supporting Information

S1 Table Table of output values from algorithm applied to artificial laboratory mixture data. (PDF)

S1 Text Accession numbers for raw data. (PDF)

S1 Fig Cut-off for low-quality samples. Number of missing SNPs for each sample in ascending order (black dots) with the threshold used for cleaning (dotted blue line).
(PDF)

S2 Fig Population structure of samples. Principal components (1–2, 1–3, 2–3) for samples and neighbor-joining tree of pairwise distance among samples both indicate limited population structure. (PDF)
