# Supplementary material for: Inferring Strain Mixture within Clinical Plasmodium falciparum Isolates from Genomic Sequence Data
Source: PLoS Comput Biol. 2016 Jun 30;12(6):e1004824. doi: 10.1371/journal.pcbi.1004824 (PMC4928962; doi:10.1371/journal.pcbi.1004824)
Supplement: S1 Table — (PDF) [file pcbi.1004824.s001.pdf]

Actual	Inferred	α	Actual	Inferred	α
(0,1)	(0,1)	1.9e-5	(0.5,0.5)	(0.52,0.48)	1.3e-5
(0.01,0.99)	(0.01,0.99)	5.3e-5	(0.6,0.4)	(0.63,0.38)	2.4e-5
(0.05,0.95)	(0.03,0.97)	1.9e-5	(0.7,0.3)	(0.72,0.28)	4.7e-6
(0.1,0.9)	(0.09,0.81)	1.4e-5	(0.75,0.25)	(0.72,0.28)	5.1e-4
(0.15,0.85)	(0.14,0.86)	1.5e-3	(0.8,0.2)	(0.77,0.23)	3.7e-6
(0.2,0.8)	(0.20,0.80)	1.0e-5	(0.85,0.15)	(0.88,0.12)	9.1e-4
(0.25,0.75)	(0.25,0.75)	1.0e-5	(0.9,0.1)	(0.90,0.10)	1.2e-5
(0.3,0.7)	(0.33,0.67)	2.8e-5	(0.95,0.05)	(0.97,0.03)	5.2e-6
(0.4,0.6)	(0.41,0.59)	1.2e-5	(0.99,0.01)	(0.99,0.01)	6.1e-5

Table S1. Table of output values from algorithm applied to artificial laboratory mixture data.
