# Supplementary material for: Inferring Strain Mixture within Clinical Plasmodium falciparum Isolates from Genomic Sequence Data
Source: PLoS Comput Biol. 2016 Jun 30;12(6):e1004824. doi: 10.1371/journal.pcbi.1004824 (PMC4928962; doi:10.1371/journal.pcbi.1004824)
Supplement: S1 Text — (PDF) [file pcbi.1004824.s002.pdf]

Supporting Information

The sample codes for the 235 samples employed in this manuscript are: PFD005, PFD008, PFD009, PFD010, PFD013, PFD014, PFD017, PFD019, PFD021, PFD022, PFD023, PFD025, PFD026, PFD027, PFD028, PFD029, PFD030, PFD032, PFD033, PFD034, PFD037, PFD038, PFD041, PFD042, PFD043, PFD044, PFD045, PFD046, PFD047, PFD048, PFD049, PFD057, PFD064, PFD065, PFD067, PFD068, PFD069, PFD071, PFD072, PFD074, PFD075, PFD079, PFD080, PFD086, PFD090, PFD091, PFD094, PFD097, PFD098, PFD101, PFD106, PFD107, PFD114, PFD115, PFD116, PFD119, PFD120, PFD121, PFD122, PFD123, PFD124, PFD128, PFD129, PFD130, PFD1307, PFD1308, PFD1309, PFD1310, PFD1312, PFD1313, PFD1314, PFD1315, PFD1316, PFD1317, PFD132, PFD1320, PFD1321, PFD1322, PFD1324, PFD1326, PFD1328, PFD1330, PFD1331, PFD1333, PFD1334, PFD1335, PFD1336, PFD1337, PFD134, PFD1340, PFD1341, PFD1342, PFD1343, PFD1344, PFD1347, PFD1348, PFD1349, PFD1350, PFD1351, PFD1352, PFD1353, PFD1356, PFD1357, PFD1358, PFD1359, PFD1360, PFD1368, PFD1369, PFD138, PFD139, PFD140, PFD141, PFD1411, PFD142, PFD1420, PFD143, PFD144, PFD145, PFD148, PFD149, PFD150, PFD151, PFD153, PFD156, PFD169, PFD172, PFD173, PFD174, PFD175, PFD177, PFD178, PFD182, PFD183, PFD184, PFD186, PFD187, PFD188, PFD189, PFD190, PFD191, PFD193, PFD194, PFD195, PFD196, PFD197, PFD199, PFD209, PFD210, PFD211, PFD212, PFD213, PFD214, PFD215, PFD216, PFD217, PFD218, PFD219, PFD220, PFD221, PFD222, PFD427, PFD446, PFD450, PFD454, PFD506, PFD507, PFD508, PFD511, PFD512, PFD513, PFD515, PFD516, PFD517, PFD519, PFD520, PFD522, PFD523, PFD527, PFD528, PFD529, PFD531, PFD534, PFD536, PFD540, PFD544, PFD547, PFD548, PFD554, PFD555, PFD557, PFD559, PFD562, PFD563, PFD564, PFD567, PFD569, PFD570, PFD573, PFD574, PFD577, PFD585, PFD586, PFD589, PFD597, PFD599, PFD601, PFD602, PFD605, PFD608, PFD609, PFD613, PFD619, PFD622, PFD630, PFD632, PFD637, PFD639, PFD640, PFD642, PFD643, PFD645, PFD648, PFD660, PFD662, PFD663, PFD664, PFD674, PFD678, PFD680, PFD682, PFD700, PFD702, PFD703, PFD705, PFD708. These data are available via the PF3K data release 3: https://www.malariagen.net/data/pf3k-3

The artificial mixture data can be downloaded from the European Nucleotide Archive https://http://www.ebi.ac.uk/ena. The accession numbers are ERS319132 ERS319134, ERS319136, ERS319138, ERS319140, ERS319142, ERS319117, ERS319120, ERS319123, ERS319126, ERS319129, ERS319131, ERS319133, ERS319135, ERS319137, ERS319139, ERS319141, and ERS319143.
