# Supplementary figures and images for: Inferring Strain Mixture within Clinical Plasmodium falciparum Isolates from Genomic Sequence Data
Source: PLoS Comput Biol. 2016 Jun 30;12(6):e1004824. doi: 10.1371/journal.pcbi.1004824 (PMC4928962; doi:10.1371/journal.pcbi.1004824)

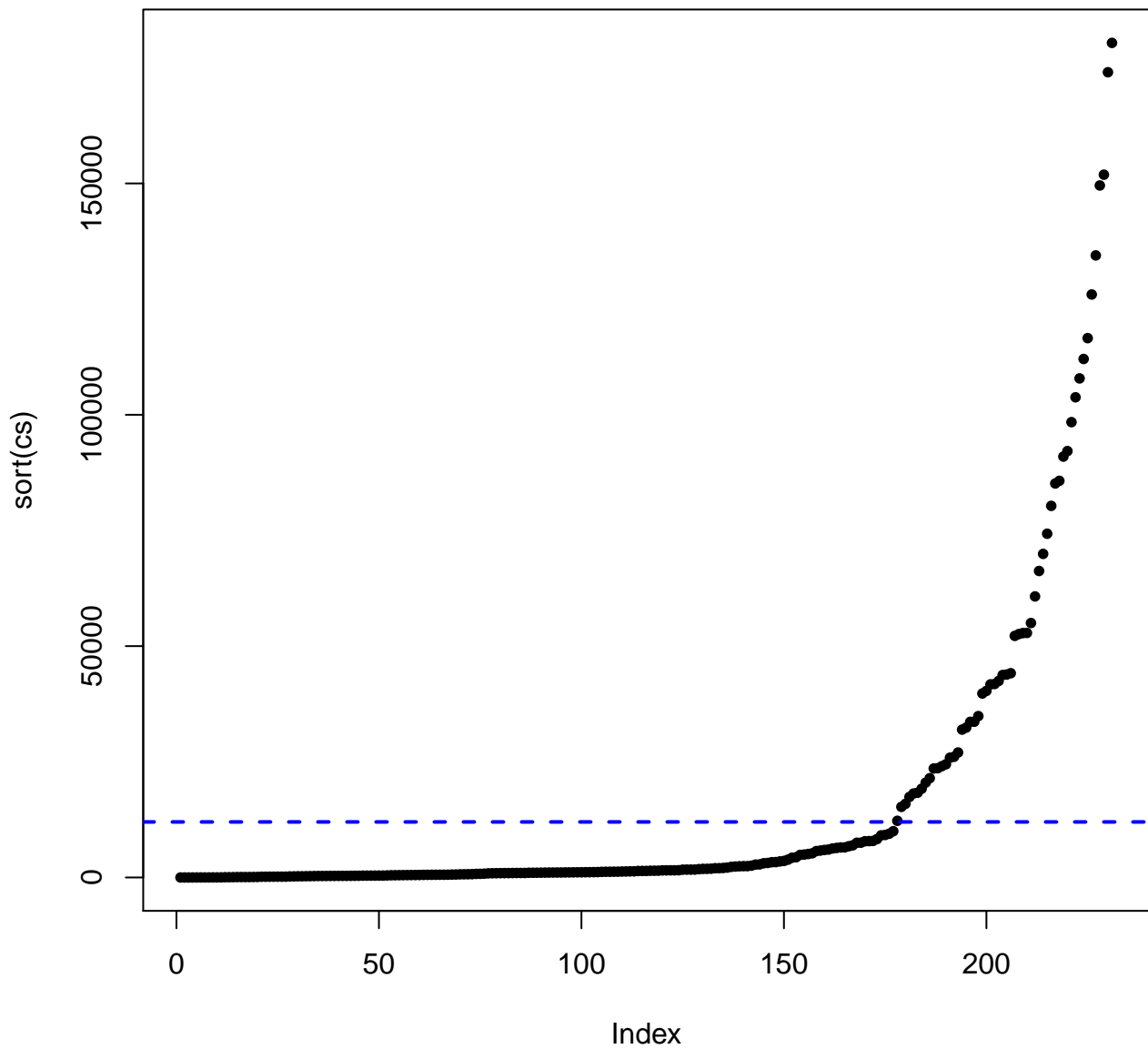

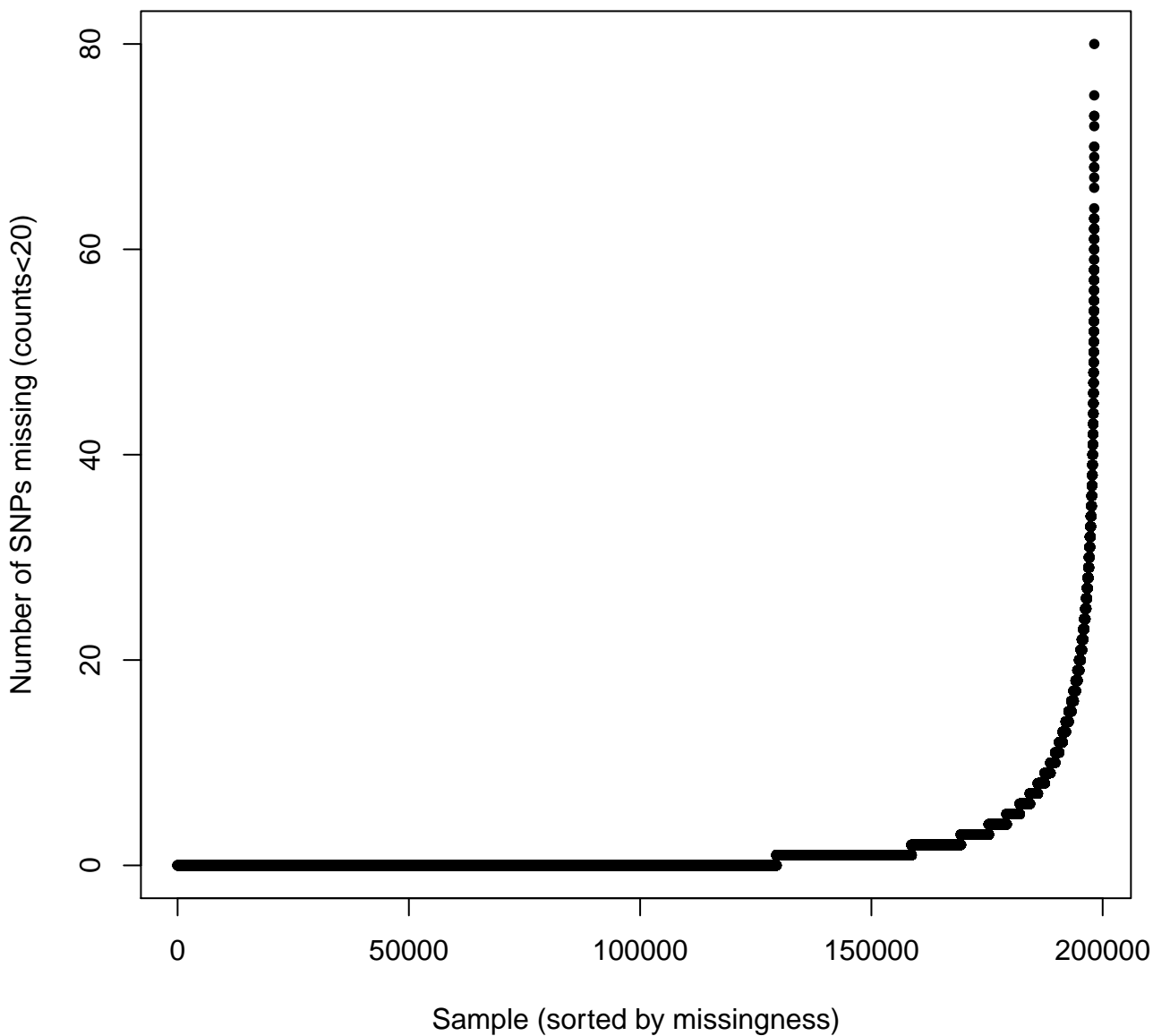

Supplement: S1 Fig — Number of missing SNPs for each sample in ascending order (black dots) with the threshold used for cleaning (dotted blue line). (PDF) [file pcbi.1004824.s003.pdf]

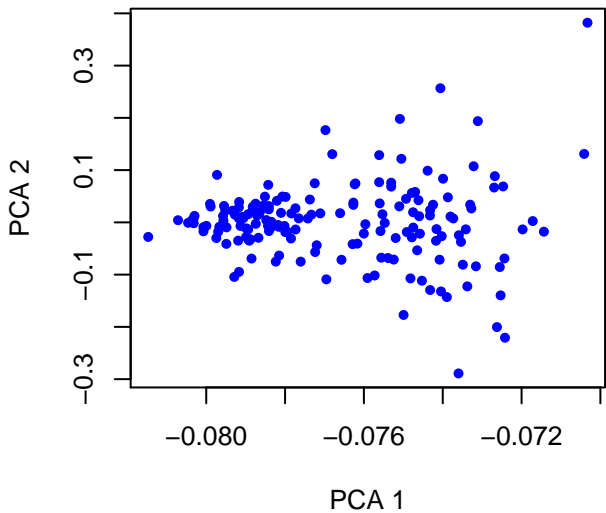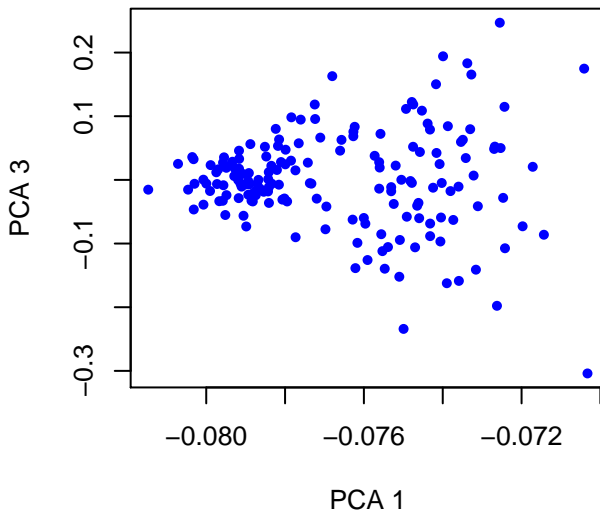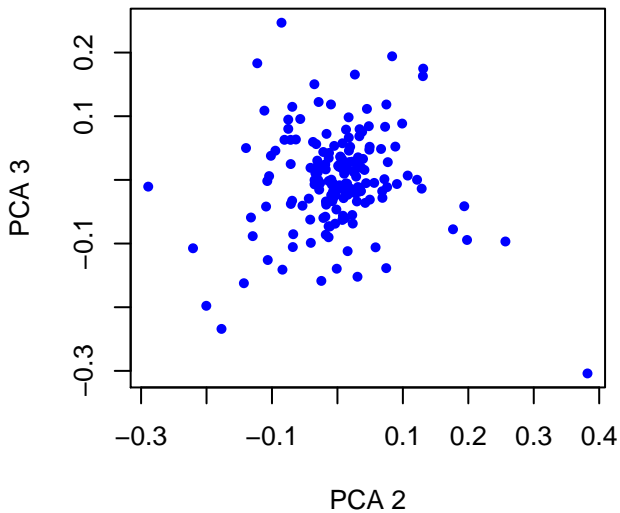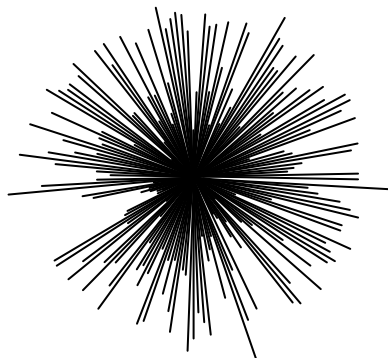

Supplement: S2 Fig — (PDF) [file pcbi.1004824.s004.pdf]
